# Future pandemics: how can we be ready?

**DOI:** 10.55730/1300-0144.5447

**Published:** 2022-05-12

**Authors:** Zeynep TÜRE, Emine ALP

**Affiliations:** 1 Department of Infectious Diseases, Faculty of Medicine, Erciyes University, Kayseri, Turkey; 2 Department of Infectious Diseases, Faculty of Medicine, Yıldırım Beyazıt University, Ankara, Turkey

**To the Editor**,

Infectious diseases have been around for as long as humans have. Pandemics have begun to appear as a result of people living together, crowded living circumstances, domestication of animals, growing grain, storing food, constructing farms and towns, and increased trade and communication. Climate change, urbanization, globalization, and increased human-animal contact have all increased the frequency of pandemics, and pandemic factors have begun to diversify [1]. We will certainly face pandemics in the future, despite the development of vaccines and preventive methods (Table).

Nevertheless, not all of the causes of pandemics can be avoided and prevented. It is critical to establish the required infrastructure in the event of a future pandemic, to take early and protective measures, to ensure rapid development in diagnosis and treatment, and to implement training programs [2]. It should be reassessed on a regular basis in light of scientific evidence, prior experience, and/or changes in national or international regulations governing infectious disease prevention and control [3].

Cities, governments, and the world must obviously prepare for future pandemics in light of the lessons learnt from the Coronavirus disease 2019 (COVID-19) pandemic.

## Lessons learnt from the COVID-19 pandemic, and precautions to take in the event of a future pandemic

### 1. Determined leadership

In any national catastrophe, planning is vital, especially in the case of a pandemic that impacts every aspect of society. Effective coordination requires lines of communication between national, regional, and local levels. The data acquired from the early detection system should be interpreted by a functioning decision-making process, and the quick actions taken should be publicly disclosed. The disease’s potential morbidity, mortality, and economic impact, as well as the expectation that these impacts will be reflected in choices taken, should be informed to the public through effective communication [4]. Different decision-making policies of world leaders have altered the impact of the COVID-19 pandemic on respective countries. South Korea, for instance, has assumed the global lead in virus containment by focusing heavily on mass testing, early contact tracing, and appropriate isolation. At the beginning of the COVID-19 pandemic, the World Health Organization (WHO) had a limited impact. In the event of future pandemics, WHO’s involvement in the global response to pandemics should be expanded by ensuring that members respond in a timely, transparent, and responsible manner [2–4].

### 2. Establishing a scientific advisory board

Trade, travel, tourism, the economy, social life, education, and a variety of other areas have all been impacted by the COVID-19 pandemic. At the beginning of the pandemic, there was a lot of misunderstanding about diagnosis and treatment algorithms, as well as isolation measures and periods. It is critical to form a scientific advisory board made up of professionals in the field from the start of the pandemic, as well as to create guides and algorithms for all necessary difficulties. Missing or unnecessary applications will be avoided in this manner [5].

### 3. Pandemic preparation planning

Pandemic simulations have been conducted in specific units such as continents, countries, hospitals, and intensive care units, based on historical outbreak experience [6]. The influenza simulation conducted by WHO revealed that pandemic preparation plans are still lacking, and that these plans should be constructed by identifying the major targets. The influenza pandemic is the focus of most simulations published in the literature. However, the late awareness of the use of face shields in the COVID-19 pandemic, as well as elements such as super contaminants, delayed the pandemic’s management. To organize pandemic preparations, several scenarios and models should be created, as well as different simulations based on transmission routes [7].

### 4. System of early warning

A powerful surveillance system is the most vital component of this system. It is crucial to promptly detect the cases in the surveillance system, along with their contacts, and implement the required isolation steps. Another aspect is the rapid detection of high-risk groups and the subsequent increase in isolation measures. These groups included people over 65 years old and those with chronic conditions during the COVID-19 pandemic. An essential factor in averting pandemics is developing rapid diagnosis systems to detect cases, establishing a strong laboratory infrastructure, and swiftly submitting positive cases to the surveillance data system. [4,8].

### 5. Competence of healthcare workers and external support to them

During the pandemic, healthcare workers are one of the occupational groups with the greatest one-on-one interaction with patients which shows up a great risk. To limit this threat, specific formal education in pandemic control, case monitoring, effective use of personal protective equipment (PPE), and isolation should be provided to healthcare staff. In addition, healthcare workers will feel safer and more motivated if they have access to quality and safe PPE’s. Healthcare workers are also physically and psychologically affected by a lack of knowledge about the disease, an insufficient supply of PPE’s, increasing physical challenges, and irregular work hours. Furthermore, compared to other public employees, healthcare workers should be well reimbursed to give psychological support for the overtime they perform [9].

### 6. Social assistance

To effectively battle the pandemic, states are enacting measures such as curfews and working time restrictions. These restrictions and regulations are having a negative impact on the economy. Women, students of all levels, and the elderly who are not allowed to leave the house, particularly service industry workers, should be provided with social, educational, and financial support. Women’s active participation in decision-making procedures makes it easier to provide the essential state benefits to women. In addition, home care services for older individuals whose social lives are restricted because of curfews should be made available. Finally, students should be given well with technological and educational assistance they require for distance learning [10].

### 7. Health-related investments

Following each pandemic, the necessity of both preventive and curative health care is realized. It is critical to build sustainable health policies, invest in primary health care and mental health, and reinforce the relation between health and social support [11].

### 8. Uniform approach to health

In order to counter the pandemic, global intervention is required. At the local, regional, and national levels, a collaborative, multisectoral, interdisciplinary approach should be formed to intervene at the global level. The protection of human, animal and plant health must be ensured by implementing a single global health strategy. Many factors should be addressed from a global perspective. These pandemic factors are developing countermeasures to pandemic risks, providing preventative health services, conducting vaccine research, and providing financial assistance to individuals who have directly affected by pandemics [10,11].

## Turkish success during the management of COVID-19 pandemic

After our country’s previous pandemic experiences, a National Pandemic Plan was published in 2006. After the 2009 influenza pandemic, it was modified [12]. This strategy was updated at the start of the coronavirus disease 2019 (COVID-19) pandemic, and it included the establishment of a scientific committee that could be accessed 24/7. The “COVID-19 Risk Assessment,” “COVID-19 Guideline”, and “Case Report Form”, as well as need-based guidelines, treatment algorithms, brochures, and other associated papers have been released in Turkey [12–14].These recommendations are revised on a regular basis based on the number of cases, current treatment procedures, mutations, and vaccinations. The World Health Organization appreciated this national fight at the beginning of the pandemic of Turkey [15].

With the beginning of the COVID-19 pandemic, the Ministry of Health organized an emergency scientific committee, and prompt and clear choices were made and implemented quickly. A strong surveillance system was used to implement a contact quarantine based on the number of cases.^1^ Healthcare workers were given assistance in implementing the protocol, identifying positive cases, and isolating contacts. Field filiation teams have been formed, and their training was kept up to date through frequent meetings. When appropriate, interventions were undertaken in accordance with the determined leadership step to limit the number of cases with completely or partially closure practices in schools or workplaces^2^. Furthermore, the COVID-19 Turkey Platform for Vaccine and Drug Development, which is administered by the Scientific and Technological Research Council of Turkey, continues to conduct vaccine and drug development research [16]. Inactivated SARS-CoV-2 vaccine has been used due to this platform. Antiviral medication research is also ongoing [17]. In scientific settings, diagnosis, treatment, case or epidemiological data are shared individually or in working groups [17].

As a conclusion, everyone has the right to a living level that is sufficient for her/his and her/his family’s health and well-being. To avoid future pandemics, preventive health care should be prioritized. To ensure national and global security, quick responses to health threats are necessary.

## Figures and Tables

**Table:** Continuing threats of new pandemics and proactive preparations for early pandemic response.

Threats	Necessary actions
The growing human population and the increasing frequency of living in megacities	Improving leadership for global and national health
The spread of zoonotic microorganisms in people living close to domestic and wild animals	Setting up a new global surveillance system
Contamination of microorganisms from animals and birds raised for nutrition to breeders and consumers	Efficient national coordination
An infection at the local epidemic level becomes a pandemic due to rapid national and international travels.	Efficient regional coordination
Insufficiency of infrastructure and hygienic conditions in situations such as wars and natural disasters	Rapid decision-making and execution
Relocation of natural habitats of vectors such as mosquitoes and ticks due to climate change	Creating cutting-edge laboratory infrastructures
Extended incubation times due to pandemic-causing microorganisms	Building strong public health infrastructure
Asymptomatic carriers	Creating physical and health conditions that are suitable for all
Mutations are seen in microorganisms, causing a pandemic.	Providing the essential financial assistance to those who have been directly or indirectly impacted by the pandemic
	Providing electronic access to a wide range of social, economic, and educational activities that can be carried out outside the home
	Facilitating access to services
	Providing mental, physical, and financial support to health care employees who work overtime

## Footnotes

1 COVID-19 toplumda salgın yönetimi rehberi. https://covid19.saglik.gov.tr/Eklenti/41940/0/covid19-toplumdasalginyonetimirehberi-19112021pdf.pdf (Accessed on February 27, 2022)

2 Güncel dönemde COVID-19 ilişkili izolasyon ve karantina uygulamaları. https://covid19.saglik.gov.tr/Eklenti/42333/0/covid-19gunceldonemdecovid19iliskiliizolasyonvekarintinauygulamalari-2pdf.pdf (Accessed on February 27, 2022)
